# Valorization of Using Agro-Wastes for Levan through Submerged Fermentation and Statistical Optimization of the Process Variables Applying Response Surface Methodology (RSM) Design

**DOI:** 10.3390/microorganisms11061559

**Published:** 2023-06-12

**Authors:** Shagufta Saeed, Sibtain Ahmed, Alina Naz, Fariha Arooj, Tahir Mehmood

**Affiliations:** 1 Institute of Biochemistry and Biotechnology, University of Veterinary and Animal Sciences, Lahore 54000, Pakistan; 2 Department of Biochemistry, Bahauddin Zakariya University, Multan 60800, Pakistan; 3 Department of Environmental Sciences, University of Veterinary and Animal Sciences, Lahore 54000, Pakistan; fariha.arooj@uvas.edu.pk; 4 Centre for Applied Molecular Biology (CAMB), University of Punjab, Lahore 53700, Pakistan

**Keywords:** levan, mango peels, *Bacillus subtilis*, submerged fermentation, response surface methodology

## Abstract

Levan is a homopolysaccharide of fructose units that repeat as its structural core. As an exopolysaccharide (EPS), it is produced by a great variety of microorganisms and a small number of plant species. The principal substrate used for levan production in industries, i.e., sucrose, is expensive and, hence, the manufacturing process requires an inexpensive substrate. As a result, the current research was designed to evaluate the potential of sucrose-rich fruit peels, i.e., mango peels, banana peels, apple peels, and sugarcane bagasse, to produce levan using *Bacillus subtilis* via submerged fermentation. After screening, the highest levan-producing substrate, mango peel, was used to optimize several process parameters (temperature, incubation time, pH, inoculum volume, and agitation speed) employing the central composite design (CCD) of response surface methodology (RSM), and their impact on levan production was assessed. After incubation for 64 h at 35 °C and pH 7.5, the addition of 2 mL of inoculum, and agitation at 180 rpm, the highest production of levan was 0.717 g/L of mango peel hydrolysate (obtained from 50 g of mango peels/liter of distilled water). The *F*-value of 50.53 and *p*-value 0.001 were calculated using the RSM statistical tool to verify that the planned model was highly significant. The selected model’s accuracy was proven by a high value (98.92%) of the coefficient of determination (R^2^). The results obtained from ANOVA made it clear that the influence of agitation speed alone on levan biosynthesis was statistically significant (*p*-value = 0.0001). The functional groups of levan produced were identified using FTIR (Fourier-transform ionization radiation). The sugars present in the levan were measured using HPLC and the levan was found to contain only fructose. The average molecular weight of the levan was 7.6 × 10^6^ KDa. The findings revealed that levan can be efficiently produced by submerged fermentation using inexpensive substrate, i.e., fruit peels. Furthermore, these optimized cultural conditions can be applied on a commercial scale for industrial production and commercialization of levan.

## 1. Introduction

Levan is one of the most significant microbial exopolysaccharides for industry. Levan is a β-(2→6) fructose polymer that possesses many distinguishing properties for industrial application such as low viscosity, strong adhesiveness, chemical- and water-holding capacities, self-assembly, good biocompatibility, nontoxicity, and high-water solubility [1]. The levan chain comprises repeated fructofuranosyl rings, coupled by β-(2, 6) linkage, that carry D-glucosyl residues at the terminal of the chain. The dominant chain of levan results from the branching of fructofuranosyl rings when coupled through β-(1, 2) linkages. This branching is essential due to its immunomodulating, antitumor, and prebiotic activities [2]. Sucrose is converted into levan by the action of the enzyme levansucrase, produced by various bacterial genera, e.g., *Bacillus*, *Halomonas*, *Pseudomonas*, *Zymomonas*, *Acetobacter*, and *Erwinia* [3].

Levan is an anticancer, anti-AIDS, anti-inflammatory, and hyperglycemic medication. It possesses other biological qualities in addition to these general properties, including adaptability, eco-friendliness, renewability, biocompatibility, and biodegradability [4]. It is a natural bonding agent and surfactant frequently used in the cosmetic industry to make skin lotions, moisturizers, and other products. As a low-caloric sweetener, levan is used in the food industry. Due to its effective properties, it also works as an antitumor agent in the medicine industry [3]. Levan produced by microorganisms is also used in food as a source of fructose and fructooligosaccharides, and serves as a fat substitute and an encasing encapsulating agent [4]. Levan is a versatile polymer with numerous uses in numerous sectors. To fulfill worldwide demand, cost-effective, large-scale levan production is essential [5]. Because of its β-(2→6) linkage, levan is soluble in water and oil. Although it does not settle out quickly in a water suspension, it has varying degrees of solubility in cold water and is soluble in hot water [6]. Levan’s intertwined branches add to its cohesive strength, and its great number of hydroxyl groups aid in forming adhesive connections with many types of substrates. This fructan is a unique polysaccharide, since it has a low intrinsic viscosity compared to other high-molecular-weight compounds. Levan has several other noteworthy characteristics, e.g., it is nontoxic and nonirritant. Levan is relatively heat stable, with a melting point of 225 °C, and is rigorously nonreducing. Levan’s exceptional qualities made it preferable as a standard film-forming agent and a stable polymer in many commercial and industrial areas [7].

Plants, yeasts, fungi, and bacteria all produce levan, and the enzyme levansucrase, also known as sucrose 6-fructosyltransferase, is used for its biosynthesis. Levansucrase is produced by a wide variety of bacterial strains; *Bacillus subtilis* may produce both inducible and constitutive extracellular levansucrase [8]. Levan-producing bacteria that have been reported in the literature include *Xhanthomonas*, *Streptococcus*, *Pseudomonas*, *Acetobacter acetigenum*, *Acetobacter pasteurianus*, *Actinomyces viscosus*, *Achromobacter* sp., *Aerobacter aerogenes*, *Bacillus amyloliquefaciens*, *Corynebacterium levaniformans*, *Gluconobacter oxydans*, *Leuconostoc mesenteroides*, *Rothis dentocariosa*, *Aspergillus versicolor*, *Aspergillus sydawi*, *Micbacterium laevaniformans*, *Corynebacterium beticola*, *Streptocuccus salivarius*, *Bacillus amyloliquefaciens*, and *Pseudomonas*. However, these bateria produce levan at a low rate [2]. According to the literature, a better yield of levan is produced by the following bacterial species: *Acetobacter pasteurianus* NRRL B-4317 (3.6 g/100 mL), *Enterobacter levanicum* NRRL B-1678 (0.7 g/100 mL), *Microbaeterium laevaniformans* ATCC 15,953 (1.2 g/100 mL), *Leuconostoc mesenteroides* sp.(1.683 g/100 mL), *Bacillus polymyxa* NRRL B-4317 (1.4 g/100 mL), *Bacillus subtilis* NRRL B-447 (1.5 g/100 mL), and *Leuconostoc mesenteroides* cremoris LF5 (2.82 g/100 mL) [9]. Various microorganisms produce r polysaccharides (levan) as a slime in the growth medium and excrete it outside the cell. Levans are also present in phlein plants but have lower molecular weight than bacterial levan. In plants, vacuole is the site where levan is produced [10]. Generally, levan produced by various organisms has the same structures but different degrees of repeating unit branches and polymerization [11].

The production of levansucrase can also occur by utilizing various agricultural remains as a substrate for fermentation in the replacement of sucrose in the media; these remains include orange peels, lemon waste, sawdust, banana waste, wheat bran, sugarcane bagasse, rice bran, maize bran, gramme bran, wheat straw, rice husk, soy hull, corncobs, tea waste, cassava waste, and apple pomace. The production rate was affected by the pH of the medium, incubation time, temperature, moisture content, and the nature of solid substrates [5]. The highest enzyme activity, 140.54 U/g from 5 g sawdust, was achieved after 72 h of incubation in 55 mL production media at pH 6 [12]. Levansucrase enzyme of approximately 62.68 U/g was produced under ideal conditions from 6 g of waste dates with an initial moisture level of 80%. Compared to sugar beet molasses and sugar cane juice, sucrose produces a high yield of levan.

The most common method for producing levan is submerged fermentation (Sm, a low-cost alternative manufacturing method). Submerged fermentation is the most widely utilized cultivation technology for generating added products. The microbial cells do not grow on the surface of the liquid media in this method, but in the liquid medium (more than 95% moisture content) with continuous stirring. This technology has several advantages including low infection risk, compactness, ease of control and automation, low labor costs, higher process reproducibility for different production cycles, and versatility in the use of a variety of biological agents such as bacteria, molds, etc. [13]. The current research was designed to select the best substrate for hyperproduction of levan by submerged fermentation using various fruit peels such as sugar cane bagasse, banana, apple, and mango peels. The statistical design of response surface methodology was used to optimize levan production conditions including agitation speed (100–200 rpm), incubation time (8–120 h), temperature (20–50 °C), and pH (5–10).

## 2. Materials and Methods

The characterized strain of *Bacillus subtilis* was obtained for levan production, and its inoculum was prepared in nutrient broth. Substrates (agricultural wastes) were collected, and their sucrose content was determined by using a polarimeter. Then, the substrates were screened to select the best levan-producing substrate, and further physicochemical parameters were optimized to achieve a high yield of levan. At the end of the process, levan was crystallized from the fermented broth and identified by FTIR. HPLC analysis was peformed to explore the sugars present in the final product, while HPSEC was used to calculate the molecular weight of the biopolymer. The details of the method are stated below.

### 2.1. Maintaining and Cultivating Microorganism

The *Bacillus subtilis* bacterial strain (FCBP-SB-0189) was acquired from the University of the Punjab, Lahore’s First Fungal Culture Bank of Pakistan. The culture of *Bacillus subtilis* was refreshed on sterilized nutrient agar plates and incubated at 30 °C for 24 h. After incubation at 30 °C, as the microbial growth appeared, the culture was preserved in the refrigerator at 4 °C [14].

### 2.2. Collection of Substrates

Various agro-wastes rich in sugar, i.e., sugar cane bagasse (*Saccharum officinarum*), banana (*Musa acuminata*), apple (*Malus domestica*), and mango peels (*Mangifera indica*), were obtained from the cafeteria of University of Veterinary and Animal Sciences (UVAS), Lahore. The fruit peels were cut into smaller pieces and dried in an oven at 60 °C for 48 h. Subsequently, the dried peels were ground into powder form and sieved (No. 18) through mesh with a pore size of 1 mm [15].

### 2.3. Inoculum Preparation

In order to prepare the inoculum, a loop full of culture was transferred to the sterile nutritional broth, shaken at 130 rpm, and maintained at a temperature of 30 °C. *Bacillus subtilis* was maintained until an OD of 0.6 at 600 nm was attained. This suspension was used as the inoculum for the fermentation experiment [16].

### 2.4. Sucrose Content Determination by Polarimeter

Fruit peel hydrolysates (10 mL) were added to 5 mL ammonia and allowed to stand for 15 min. The sample was neutralized by adding acetic acid. Then, 12 mL of zinc acetate was added, and filtration was carried out for the precipitation of proteins and fats. Using an analyzer (Manual Polarimeter APD 440), the angle of rotation of sucrose was observed, and this was used to determine its quantity [17].

### 2.5. Pretreatment of Substrates

Each substrate (5 g) was added to 100 mL of distilled water, autoclaved at 121 °C for 10 min, and filtered through filter paper [18].

### 2.6. Choosing the Optimal Substrate for the Manufacture of Levan

Each fruit peel hydrolysate (25 mL) was placed in a 250 mL flask after adjusting pH 7 (using 1 N HCl and 1 N NaOH), autoclaved, and 2 mL of inoculum of *Bacillus subtilis* was added to each flask. After adding inoculum, all the flasks were placed at 150 rpm in a shaking incubator at 30 °C for 48 h to produce levan. The substrate that yielded the highest levan production was subjected to a further optimization process [19].

### 2.7. Process Optimization for Greatest Production of Levan

Utilizing the central composite design (CCD) of response surface methodology (RSM), several cultural conditions (temperature, incubation time, pH, inoculum volume, and agitation speed) were then optimized to provide the highest yield of levan after choosing the best substrate, as indicated in Table 1. The following parameters were utilized to optimize levan production: incubation period (8–120 h), pH (5–10), temperature (20–50 °C), inoculum volume (1–5 mL), and agitation speed (100–200 rpm). In total, 32 experiments were carried out in triplicate with different parameters to express the interaction between them and measure their combined and individual effects on levan production using response surface methodology [20].

### 2.8. Precipitation and Quantification of Levan

The fermented media was removed from the flask and centrifuged for 20 min at 6000 rpm. Then, 10 mL of supernatant was used for further extraction, and an organic solvent, such as ethanol, was added twice for precipitation. Precipitation was carried out overnight at 4 °C. Subsequently, centrifugation was carried out again at 6000 rpm for 20 min. The precipitated pellet was washed twice more with distilled water [21]. After the extraction of levan, precipitates were hydrolyzed with 1 mL of 0.1 M HCl and the fructose content was estimated by the DNS method. The quantity of fructose found in the sample was divided by the factor 1.11 to determine the quantity of levan produced [22].

### 2.9. Characterization of Levan

#### 2.9.1. Fourier-Transform Infrared (FTIR) Spectroscopy

Using Fourier-transform ionization radiation (FTIR) spectroscopy (Shimadzu/Prestige-21), the chemical bonds and functional groups of the levan synthesis were determined. Sample of levan produced (2 mg) was mixed with 200 mg of KBr, dried and ground. Then, the mixture was squeezed to form transparent pellets, placed in the cell with skewers, and absorption spectrum was collected.

#### 2.9.2. Analysis of Sugar Content with High-Performance Liquid Chromatography (HPLC)

Each component of the mixture was dispersed, identified, and quantified using high-performance liquid chromatography (Shimadzu RID-10A). An NH_2_ column, at 3 µm particle size (50 × 4.6 mm) and equipped with refractive index detector, was used for the procedure. The mobile consisted of acetonitrile: deionized water (50:50) run at a flow rate of 1 mL/min, a temperature of 40 °C, and an injection volume of 20 µL. Standard solutions of fructose, glucose, and xylose (25 µg/mL) were prepared using mobile phase. Hydrolysed sample of levan was also prepared accordingly [23].

#### 2.9.3. Using High-Performance Size-Exclusion Chromatography (HPSEC) to Calculate the Molecular Weight of Levan

The molecular weight of levan was determined by high-performance size-exclusion chromatography (HPSEC). In order to carry this out, 2 TSK-GEL columns in series were used while 0.1 M NaNO_3_ was used as the eluent at a flow rate of 0.6 mL/min. Levan at a concentration of 5 mg/mL was filtered via a 0.22 µm filter before being injected into the columns, which were kept at a constant temperature of 25 °C. The standards were commercial pullulans with various molecular weights [11].

## 3. Results and Discussion

### 3.1. Analysis of Sucrose Content of Fruit Peels

Sucrose is the primary carbon source utilized by industries for the formation of levan. As a result, a polarimeter was used to assess the sucrose content of different fruit peels, and the results are shown in Table 2.

### 3.2. Screening of Best Substrate for Levan Production

**Figure 1:**
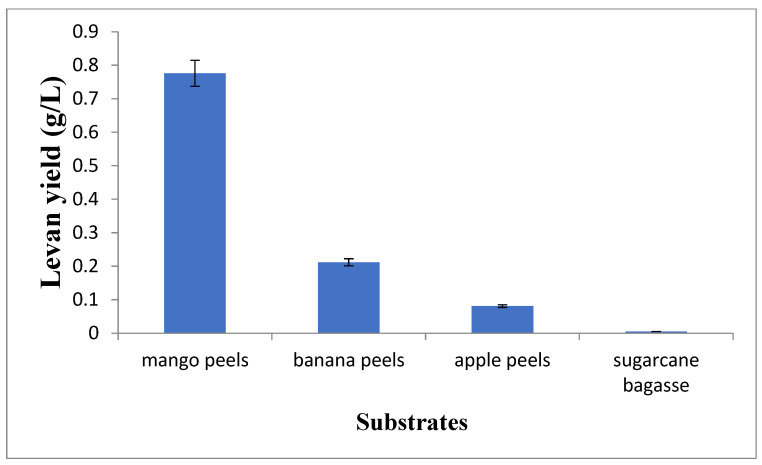
Screening of substrates for levan production using submerged fermentation.

For the production of levan, *Bacillus subtilis* was cultured for 24 h in a fermentation medium containing the following substrates at a fruit-to-peel ratio of 1:5: *Mangifera indica* (mango), *Musa acuminate* (banana), *Malus domestica* (apple), and *Saccharum officinarum* (sugarcane bagasse). After incubation, all substrates were analyzed separately for levan production. The highest levan production (0.776 g/L) was observed for *Mangifera indica* peels, followed by *Musa acuminate* peels (0.212 g/L), *Malus domestica* peels (0.081 g/L), and *Saccharum officinarum* (0.0054 g/L) by submerged fermentation, as shown in Figure 1. As the main substrate for levan production is sucrose, the highest levan production was observed using *Mangifera indica*, which has the highest sucrose content (as shown in Table 2) among the different fruit peels tested.

### 3.3. Levan Biosynthesis Optimization by RSM

Using mango peel hydrolysate as a substrate for submerged fermentation, CCD (RSM) was used to optimize the production parameters for levan biosynthesis. The trial series and value of independent variables for levan production are shown in Table 1 and Table 3. Biosynthesized levan (g/L) was considered the dependent variable, whereas incubation duration, inoculum size, temperature, pH, and agitation speed were chosen as the independent factors. The highest yield of levan (0.717 g/L) was attained after 64 h of incubation at pH 7.5, temperature 35 °C, with 2 mL of inoculum, and an agitation speed of 180 rpm. The minimum yield (0.013 g/L) of levan was attained at an incubation time of 72 h, pH 7.5, a temperature of 50 °C, with 2 mL inoculum, and at a shaking speed of 100 rpm (Table 3).

The output of statistical results, as shown in Table 3, with an *F*-value of 50.53 and a *p*-value of 0.001, confirms the importance of the suggested model. The coefficient of determination (R2) shows a value of 98.92%, indicating that the model is accurate and precise. If the *p*-value is less than 0.05, the impact of independent variables is considered significant. It was clear from the results obtained from ANOVA, stated in Table 4, that the impact of agitation speed (Factor E) is significant on levan biosynthesis, while all other parameters had an insignificant effect.

The regression model equation is shown as:

Levan (g/L) = 2.554 − 0.00949 A − 0.0653 B − 0.2274 C + 0.1934 D − 0.00904 E − 0.000002 A*A

+ 0.000253 B*B + 0.00500 C*C + 0.00397 D*D + 0.000006 E*E + 0.000083 A*B

+ 0.000241 A*C − 0.000308 A*D + 0.000037 A*E + 0.001676 B*C − 0.00150 B*D

+ 0.000249 B*E − 0.00679 C*D + 0.000710 C*E − 0.000780 D*E

**Figure 2:**
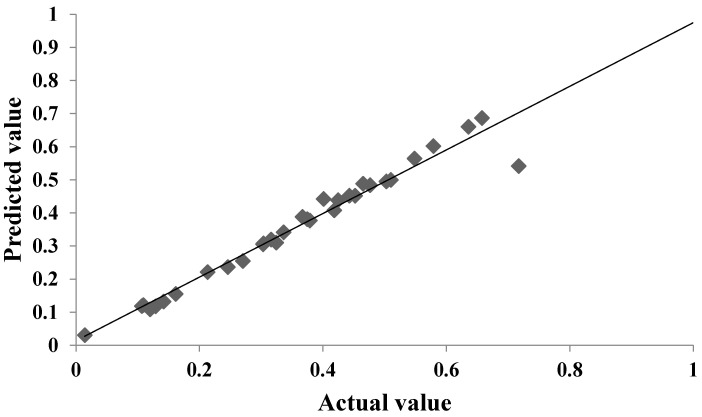
Comparison of the levan production graph between observed and expected values.

**Figure 3:**
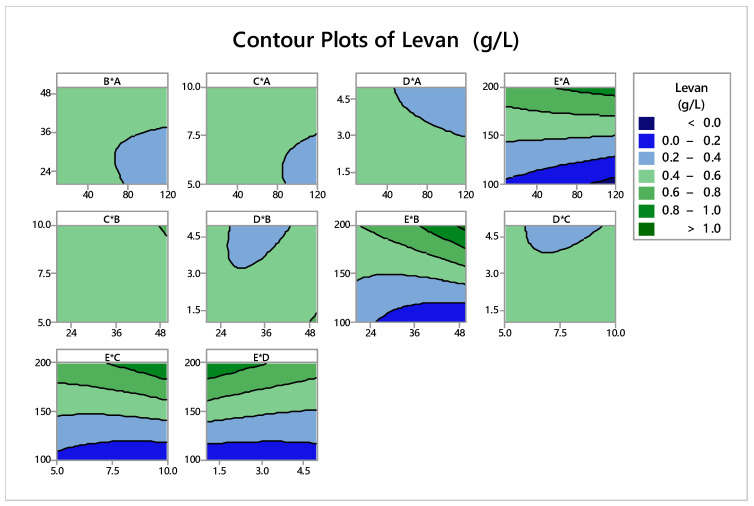
Contour plots for levan production from CCD show the interactions of various response factors.

The graphic illustration of observed and anticipated measurements of levan production is shown in Figure 2. The observed values were discovered to be rather close to the expected ones. The contour plots in Figure 3 indicate the interactions between various independent factors for levan formation. In these graphs, there is a link between two independent variables and a third dependent variable. The color changes demonstrate the stages of levan generation that coexist between two independent components while maintaining a constant value for the third element.

**Figure 4:**
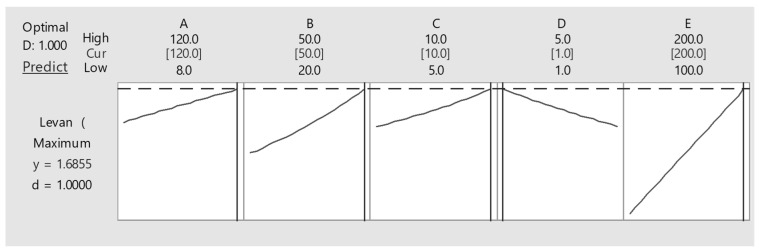
Desirability chart for levan production using mango peels via submerged fermentation.

RSM was used to create the desirability chart for levan production, as indicated in Figure 4. It showed that the highest production expected for levan synthesis would be 1.68 g/L if the values of parameter A are 120 h, parameter B is 50 °C, parameter C is 10, parameter D is 5 mL, and parameter E is 200 rpm; however, in the actual experiment, the greatest quantity of levan (0.717 g/L) was attained when parameter A was 64 h, parameter b was 35 °C, parameter C was pH 7.5, parameter D was 2 mL of inoculum, and parameter E was 180 rpm. When compared, this reaction approached the predicted values, confirming the planned model’s accurate prediction.

### 3.4. Identification, Quantification, and Characterization of Levan

#### 3.4.1. FTIR

**Figure 5:**
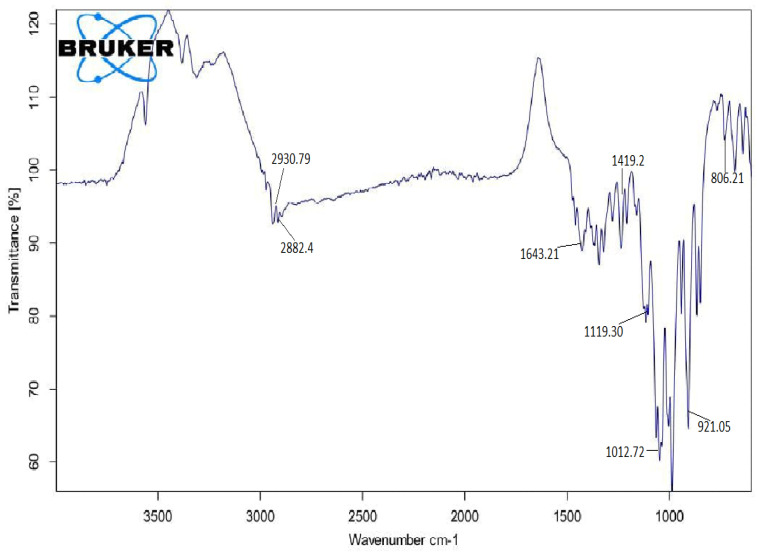
FTIR spectrum of levan.

FTIR analysis was used to identify the levan precipitates formed after optimizing the parameters, as shown in Figure 5. Levan was present in the fermented sample, as evidenced by a pattern of peaks in the FTIR spectrum of the material produced by microbial fermentation. C-H stretching vibration was observed at 3000–2800 cm^−1^ (especially at 2930.79 and 2882.4 cm^−1^). The methylene group was present at 2930.79 cm^−1^, where asymmetrically vibration was observed. Methylene group bending vibrations were observed at 1419.2 cm^−1^. Because of the bound water, a wide band was observed at 1643.21 cm^−1^. CO-H stretching vibration first occurred at 1119.30 cm^−1^. A strong, broad peak at 1012.72 cm^−1^ was considered glycosidic linkage (C-O-C) stretching vibration in pyranose or furanose, which is a carbohydrate characteristic. At 921.05 and 806.21 cm^−1^, broad peaks appeared, which was interpreted to be furanose of sugar units. According to the literature, the characteristic peaks of several pyranoses are indicated in FTIR spectra as 2 broad peaks at 918 and 769 cm^−1^. [24]. In general, several factors, including strain type, fermentation conditions, medium composition, and extraction procedure, have an impact on polysaccharide molecular weight. Levan’s typical molecular weight was found to be 7.6 × 10^6^ KDa. Wang et al. [25] found the average molecular weight of levan to be 1.56 × 10^6^ Da. A study by Carbajal et al. [26] found that high-molecular-weight levan (2.3 × 10^6^ Da) and low-molecular-weight levan (7.2 × 10^3^ Da) were both present in levan generated by *Bacillus subtilis.* Xu et al. [27] found that microbial levans have a wide range of molecular weights, depending on the producers and growth conditions. B. subtilis created levans with molecular weights of 11 kDa and 1800 kDa, whereas levan with a molecular weight of about 2106 Da were produced by *B. polymyxa* (NRRLB-18475).

#### 3.4.2. HPLC Analysis

An HPLC chromatogram indicated that the levan we produced contained only fructose sugar, when compared with the chromatogram of standard levan containing three different sugars (fructose, glucose, and xylose).

## 4. Discussion

The entire budget for fermentation is determined by the substrate used in the manufacturing process. Sugar, i.e., sucrose, is commonly used as a carbon source for levan production, which increases the cost of the fermentation process. Pakistan is an agro-based country generating a large amount of agro-waste each year which pollutes the environment as it cannot be utilized further. Similarly, numerous food processing firms produce numerous sugar-rich byproducts that have no practical purpose. Agricultural waste can be used in bioconversions to produce cost-effective products and assist in reducing pollution caused by agro-waste residues [28]. There is an increasing trend in biotechnology to recycle sugar-rich waste into commercially viable substances such as levan. SSF fermentation is usually used for levan production, but it produces a low yield. As a result, submerged fermentation, using waste fruit peels for the production of levan, was applied in this study [19]. Additionally, RSM has been applied as a statistical design for maximizing a number of factors for levan yield. It is useful for estimating how various manufacturing process variables interact. In the present study, mango peels were used as the substrate, and SmF was used to achieve the greatest levan production (0.717 g/L) at 2 mL inoculum size, 7.5 pH, 180 rpm agitation speed, and 35 °C temperature. According to the cited literature, sucrose is the best levan production substrate. Therefore, sucrose-based substrates, such as fruit byproducts and agricultural wastes, are employed for the microbial synthesis of levan. Kanakdande et al. [15] reported levan production using banana peels (0.50 mg/mL) and orange peels (0.58 mg/mL) for 72 h of incubation, at 37 °C and pH 7. Han et al. [11] produced levan using substrates such as sucrose, sugarcane juice, and sugar beet molasses. They claimed that 100 mL of a growth medium contained 15% sucrose and 3.6 g of levan. In contrast to sucrose, the yields of levan from sugarcane juice (0.65 g/L yield) and beet molasses (0.36 g/L yield) were very low. When fermentation conditions were 48 h and pH 6.0, Nasab et al. [29] discovered that 1.048 g of levan per 100 mL of date juice was produced compared with 48.9 g/L levan production recovered from culture media containing sucrose (20%). SmF produces more levan than solid state fermentation. This is because SSF does not continuously combine nutrients and bacteria (due to lack of continuous agitation), although the method has the benefit of being a low-cost procedure.

The current study adjusted the incubation period from 8 to 120 h to produce a greater amount of levan. A greater production of levan was observed at 64 h of incubation time. However, the effect of incubation time was observed to be insignificant in this study. Product inhibition is responsible for reducing levan production after 72 h of incubation, because the enzyme level decreased with time, which may have been caused by moisture loss or denaturation of the enzyme brought on by pH changes during fermentation [5]. According to a study by Xavier et al. [30], the highest amount of levan was produced by *Bacillus licheniformis* ANT 179 utilizing a sucrose-rich medium, including sugarcane juice, after 48 h of incubation. Contrary to the current study, Qaysi et al. [31] reported that *P. agglomerans* ZMR7 produced the highest amount of levan after 96 h of incubation. Chidambaram et al. [32] reported that the highest levan production was observed by using Bacillus *subtilis* MTCC 441, with 20 h of incubation, and using sucrose as the sole carbon source. Shih et al. [33] reported that the highest levan production from *Bacillus subtilis*, under optimal conditions, was observed after 48 h of incubation with 250 g/L of available sucrose. In another study, Santos et al. [34] used *B. subtilis* CCT 7712 isolated from natto and produced the highest yield of levan from 400 g/L of sucrose in 16 h. The time for production depends on the microorganism and substrate used in the experiment.

Fermentation was carried out at pH ranging from 5 to 10 to optimize levan production by RSM. The current study achieved the highest levan production at pH 7.5, but its overall effect was insignificant, according to ANOVA analysis. Each microbe has a pH range for development and activity, with an ideal growth within the range. Therefore, pH range depends on the microorganism. Reduced growth and product formation was the outcome of raising or lowering pH. The enzyme’s activity was controlled by the ionization state of its amino acids, which was influenced by the pH of the medium to which it was exposed [5]. Erkorkmaz et al. [35] expressed identical findings, indicating that *H. smyrnensis* AAD6T reached its greatest biomass and levan titer at an initial pH value of 7.0. Moussa et al. [16] experimented to determine the optimal pH for levan synthesis using several values (7, 7.5, 7.8, 8, and 8.5). When the pH was elevated from 7 to 7.8, *B. phenoliresistens* steadily produced more levan. *B. phenoliresistens* produced 7.88 g/L of levan at pH 7.8, the highest level. Nasab et al. [29] determined that the ideal pH for manufacturing levan was 6.0 by observing the effects of pH on the yield of the purified levan. The pH of the medium declined from 7.0 to around 6.8 over the course of 24 h of fermentation. In contrast to current findings, Shih et al. [33] reported pH 6 as the optimum pH for levan synthesis by *Bacillus subtilis*. Taleb et al. [36] also reported significantly different results and stated that levan production increased progressively as the pH values rose from 5.5 to 6.5, peaking at the initial pH of 6.5. Levan yield was shown by Hou et al. [37] to be stable between pH 4 and 7.5, with *Bacillus subtilis* being used to provide the highest yield at pH 6.5. Fermentation was carried out at different temperatures ranging from 20 to 50 °C to study the influence of incubation temperature on levan biosynthesis. At 35 °C, the greatest levan production was observed 0.717 g/L. Because levansucrase lost its ability to create levan beyond 50 °C but retained its ability to hydrolyze sucrose at this temperature, temperatures above this range may cause less levan to be produced. This might be a result of the temperature having an inhibiting influence on enzyme activity and making it less stable [5]. The findings by Goncalves et al. [38] support our findings—that the optimum temperature for levan formation is between 25 and 37 °C. Wu et al. [39] also showed that the greatest yield of levan was observed at 35–40 °C; the yield gradually dropped at higher temperatures. By employing *Bacillus subtilis*, they discovered that the influence of temperature on levan production might indicate that the levansucrase’s levan-producing activity peaks at 37 °C. Ramya et al. [40] found that the optimum temperature for levan yield was between 30 and 35 °C, using *Bacillus subtilis* at a sucrose concentration of 250 g/L. Tieking et al. [41] reported outcomes dissimilar to our results; in their study, the temperature required for levan synthesis varied from one organism to the other. *Zymomonas mobilis* was used to investigate the amount of levan produced at various temperatures and they discovered that the greatest concentration of levan, 27.2 g/L, was obtained at 25 °C. The least amount of levan formation was found at temperatures ranging from 35 °C to 40 °C. To determine the optimum temperature for levan formation, Khudair et al. [21] selected the isolate *L. mesenteroides* spp. *cremoris* (LF5) and incubated it at 25, 30, and 35 °C temperatures. The optimum temperature for levan production was 30 °C, with a yield of 1.482 g/100 mL (7.4%) at this temperature. Kang et al. [42] investigated the expression and cloning of levansucrase in *E. coli* and discovered that 30 °C was the ideal temperature for levan formation. The generation of levansucrase, which is important for levan formation by *Zymomonas mobilis*, is inhibited at high fermentation temperatures of 30 °C to 42 °C. Santos et al. [43] used 30 °C temperature to manufacture biopolymer levan from *Z. mobilis*.

The impact of inoculum size and levan production was also examined in the current study using the statistical design of RSM. The inoculum size of 2 mL produced the highest yield of levan at 35 °C, but its effect was found to be insignificant according to ANOVA analysis. Compared to smaller inoculum sizes, larger inoculum sizes may deplete the nutrients faster in the fermentation media. For levan production, Shih et al. [44] utilized a 5% (*v*/*v*) inoculum of *B. subtilis* (natto) Takahashi. The effect of inoculum size on levan production was also scrutinized by Khudair at al. [21]. *L. mesenteroides* ssp. (LF5) was inoculated with different sizes of inoculum (2, 4, 6, 8, and 10%). According to the findings, the optimal inoculum size for levan generation was 4% (1.810 g/100 mL). At 4% inoculum size, Yang et al. [17] successfully isolated polysaccharides from *Cordyceps militaris*.

Agitation speed was varied from 100 to 200 rpm, and its effect on levan production was optimized by using the central composite design of RSM. The greatest levan production (0.717 g/L) occurred at 180 rpm at 35 °C (Table 3). The results of Laddah et al. [45] did not agree with ours; they examined the effect of agitation on levan production by using 100 g/L sucrose, 300 °C for 48 h on *Bacillus subtilis* (KC243314). At 50 rpm, levan output was 28.0 g/L. When agitation speed was raised to 100 rpm, levan production was increased by 30.6 g/L. Berekaa et al. [46] also reported that agitation speed had a direct proportionality with levan production, with the highest value (15.0 g/L) at 100 rpm and the lowest value (1.2 g/L) at 250 rpm. With *Bacillus subtilis* NATTO, Santose et al. [43] achieved a maximum levan output of 34 g/L at 150 rpm. The results are consistent with those of Shih et al. [47], who discovered that the optimal shaking speed was between 150 and 200 rpm. As indicated in the literature, the greatest levan production was obtained when agitation speed was set from 100 to 200 rpm.

## 5. Conclusions

Many factors were studied, including the impact of the incubation period, pH, temperature, inoculum size, and agitation speed. Fruit peels were effectively used as the substrate for submerged fermentation that successfully produced levan. The greatest yield (*p*-value ≤ 0.05) of levan (0.717 g/L after 64 h of incubation at pH 7.5, 35 °C, and 180 rpm) was obtained using mango peel hydrolysate as the substrate. These optimized cultural conditions can be further applied on a pilot scale (in bioreactor studies) to enhance the productivity of levan to meet the industrial requirements of biopolymer.

## Figures and Tables

**Table 1:** Level of independent factors in planned experiment.

Codes	Independent Parameters	Unit	Low Level	High Level
A	Incubation time	h	08	120
B	Temperature	°C	20	50
C	pH	-	05	10
D	Inoculum volume	mL	01	5
E	Agitation speed	rpm	100	200

**Table 2:** Sucrose content of different fruit peels.

Substrates	Sucrose Content (g/L)
*Mangifera indica* (mango) peels	89.11 ± 0.54
*Musa acuminate* (banana) peels	29.77 ± 0.32
*Malus domestica* (apple) peels	14.02 ± 0.41
*Saccharum officinarum* (sugar cane) bagasse	17.76 ± 0.18

**Table 3:** CCD for maximizing the production of levan from mango peels.

						Levan (g/L)		
Experiment	A	B	C	D	E	Observed	Predicted	Residuals
1	48	30	7.5	1	150	0.401	0.442	−0.041
2	64	25	7.5	2	130	0.336	0.341	−0.005
3	120	30	5	1.5	150	0.336	0.388	−0.021
4	48	30	8	3	130	0.315	0.319	−0.003
5	48	35	7	5	200	0.635	0.660	−0.024
6	48	30	5	2	150	0.476	0.483	−0.007
7	120	30	7	5	180	0.464	0.487	−0.022
8	48	40	7	4.5	100	0.128	0.117	0.010
9	24	25	10	3	180	0.578	0.602	−0.023
10	72	50	7.5	2	100	0.013	0.030	−0.016
11	24	30	7	1	130	0.374	0.381	−0.006
12	24	35	7.5	2.5	150	0.424	0.438	−0.013
13	12	40	8	3.5	130	0.303	0.308	−0.004
14	24	50	5	4	100	0.141	0.132	−0.009
**15**	**64**	**35**	**7.5**	**2**	**180**	**0.717**	**0.541**	**0.175**
16	64	50	6	2.5	110	0.106	0.118	−0.012
17	64	30	5	4	150	0.451	0.451	0.0003
18	48	25	7	4	120	0.324	0.310	0.013
19	12	25	7.5	5	150	0.509	0.499	0.010
20	72	40	6	4.5	110	0.161	0.155	0.006
21	8	30	7.5	1.5	100	0.245	0.236	0.009
22	72	25	5	2	130	0.378	0.376	0.001
23	24	30	5	3	130	0.418	0.407	0.010
24	72	20	10	3.5	130	0.302	0.305	−0.002
25	24	25	7	4.5	150	0.502	0.495	0.007
26	72	35	5	4.5	180	0.548	0.564	0.015
27	24	50	6	5	100	0.119	0.108	−0.012
28	120	20	6	5	120	0.270	0.254	−0.009
29	48	50	7	2.5	110	0.108	0.121	−0.029
30	12	35	7.5	3	150	0.442	0.452	−0.008
31	48	35	6	3.5	200	0.657	0.686	−0.06
32	8	25	10	1	100	0.213	0.221	−0.14

The bold value denotes the highest value for high-yield output.

**Table 4:** Analysis of variation for mango peel-derived levan biosynthesis.

Source	DF	Adj SS	Adj MS	*F*-Value	*p*-Value
Model	20	0.955516	0.047776	50.53	0.001
Linear	5	0.355740	0.071148	75.24	0.001
A	1	0.000234	0.000234	0.25	0.629
B	1	0.001084	0.001084	1.15	0.307
C	1	0.000671	0.000671	0.71	0.418
D	1	0.004332	0.004332	4.58	0.056
E	1	0.129846	0.129846	137.32	0.000
Square	5	0.004979	0.000996	1.05	0.435
A*A	1	0.000055	0.000055	0.06	0.815
B*B	1	0.001448	0.001448	1.53	0.242
C*C	1	0.001383	0.001383	1.46	0.252
D*D	1	0.000399	0.000399	0.42	0.530
E*E	1	0.000369	0.000369	0.39	0.545
Two-Way Interaction	10	0.026064	0.002606	2.76	0.056
A*B	1	0.002037	0.002037	2.15	0.170
A*C	1	0.000875	0.000875	0.93	0.357
A*D	1	0.001883	0.001883	1.99	0.186
A*E	1	0.008161	0.008161	8.63	0.014
B*C	1	0.002803	0.002803	2.96	0.113
B*D	1	0.001176	0.001176	1.24	0.289
B*E	1	0.008845	0.008845	9.35	0.011
C*D	1	0.001108	0.001108	1.17	0.302
C*E	1	0.007263	0.007263	7.68	0.018
D*E	1	0.009148	0.009148	9.68	0.010
Error	11	0.010401	0.000946		
Total	31	0.965918			

## Data Availability

The publication contains the data used to support the study’s findings.
